# Cell-cycle dependent localization of MELK and its new partner RACK1 in epithelial *versus* mesenchyme-like cells in Xenopus embryo

**DOI:** 10.1242/bio.20136080

**Published:** 2013-08-21

**Authors:** Isabelle Chartrain, Yann Le Page, Guillaume Hatte, Roman Körner, Jacek Z. Kubiak, Jean-Pierre Tassan

**Affiliations:** 1 UMR 6290 CNRS Institut de Génétique et Développement de Rennes – Université de Rennes 1, Cell Cycle Group, SFR Biosit, 2 Avenue du Professeur Léon Bernard, CS 34317, 35043 Rennes Cedex, France; 2 Max-Planck-Institute of Biochemistry, D-82152 Martinsried, Munich, Germany; * Present address: Institut de Recherche en Santé, Environnement et Travail (IRSET), 35042 Rennes, France

**Keywords:** Cell division, Cell polarity, Development, Tight junction

## Abstract

Maternal Embryonic Leucine zipper Kinase (MELK) was recently shown to be involved in cell division of Xenopus embryo epithelial cells. The cytokinetic furrow of these cells ingresses asymmetrically and is developmentally regulated. Two subpopulations of xMELK, the mMELK (for “mitotic” xMELK) and iMELK (“interphase” xMELK), which differ in their spatial and temporal regulation, are detected in Xenopus embryo. How cells regulate these two xMELK populations is unknown. In this study we show that, in epithelial cells, xMELK is present at a higher concentration at the apical junctional complex, in contrast to mesenchyme-like cells, which have uniform distribution of cortical MELK. Interestingly, mMELK and iMELK also differ by their requirements towards cell–cell contacts to establish their proper cortical localization both in epithelial and mesenchyme-like cells. Receptor for Activated protein Kinase C (RACK1), which we identified as an xMELK partner, co-localizes with xMELK at the tight junction. Moreover, a truncated RACK1 construct interferes with iMELK localization at cell–cell contacts. Collectively, our results suggest that iMELK and RACK1 are present in the same complex and that RACK1 is involved in the specific recruitment of iMELK at the apical junctional complex in epithelial cells of Xenopus embryos.

## Introduction

MELK (Maternal Embryonic Leucine zipper Kinase) is a serine/threonine protein kinase of evolutionary conserved KIN1/PAR-1/MARK family. Kinases belonging to this family of proteins are found from yeast to human and are involved in diverse functions such as cell polarity and cell cycle control (Tassan and Le Goff, 2004). MELK regulates neural progenitor cell renewal (Nakano et al., 2005), apoptosis (Jung et al., 2008; Lin et al., 2007), mRNA splicing (Vulsteke et al., 2004), haematopoiesis (Saito et al., 2005) and asymmetric cell division (Cordes et al., 2006).

MELK has emerged as a potentially important therapeutic target in the field of cancer research. Indeed, several studies have shown that MELK expression is dramatically increased in cancers of various tissue origins (Gray et al., 2005; Marie et al., 2008; Nakano et al., 2008). Moreover, a direct correlation between high MELK expression and malignancy grade has been reported in melanoma (Ryu et al., 2007), breast cancer (Pickard et al., 2009) and brain tumors (Marie et al., 2008; Nakano et al., 2008). This, together with the data showing a decrease in cell proliferation of some cancer cell lines after MELK knockdown by siRNA, has led to the hypothesis that the high levels of MELK activity may provide an advantage to tumor cells. In addition, MELK involvement in the inhibition of apoptosis may also promote tumor cell survival (Lin et al., 2007). Increased MELK expression is associated with poor prognosis in breast cancer (Pickard et al., 2009). Thus, MELK could also be a potentially important prognosis marker for some types of cancers. Interestingly, it has recently been shown that the antibiotic siomycin A decreased MELK expression and correlatively inhibited renewal of brain cancer derived stem like cells *in vitro* and a glioblastoma tumor growth *in vivo* (Nakano et al., 2011). Although MELK appears to be a good candidate for the development of future diagnosis tools and anticancer drugs, its precise function remains unclear.

Recently, we have shown that Xenopus MELK (xMELK) is involved in embryonic cell division (Le Page et al., 2011). MELK expression is tightly regulated during early embryogenesis in Xenopus, where it was initially identified under the name of Eg3 (Paris and Philippe, 1990), and in the mouse (Heyer et al., 1997). In contrast, in adults, the expression of MELK is limited to cells engaged in cell cycle progression and is undetectable upon cell differentiation (Badouel et al., 2010). In human cells and Xenopus embryos, MELK is phosphorylated during mitosis, which correlates with the increase in its catalytic activity (Blot et al., 2002; Davezac et al., 2002). In xMELK, we have identified multiple sites phosphorylated specifically during mitosis (Badouel et al., 2006). The two major mitotic kinases, cyclin B-CDK1 complex and mitogen-activated protein kinase ERK2, participate in these phosphorylation events and enhance MELK activity *in vitro*. Thus, mitosis appears critical in the regulation of MELK activity, and conversely MELK may regulate mitotic progression.

Consistent with this specific regulation during mitosis, we have shown, using xMELK knockdown and overexpression, that this kinase is involved in the control of cytokinesis in Xenopus embryos (Le Page et al., 2011). xMELK associates with anillin which acts as a platform for the assembly of proteins involved in cytokinesis such as myosin and RhoA small GTPase. In early embryos, xMELK, as well as other cytokinetic proteins including anillin, becomes highly concentrated at the division furrow shortly prior the onset of cytokinesis (Le Page et al., 2011). The localization of xMELK at the division furrow is a dynamic event, which correlates with a conformational rearrangement of the molecule and is regulated during early development. In dividing cells, from the first embryonic cleavage up to the blastula stage, the xMELK is concentrated at the cell cortex in an equatorial band, which ultimately corresponds to the cytokinetic furrow. However, at the later developmental stage (in gastrula), the xMELK is neither concentrated in the equatorial band nor at the cytokinetic furrow. Our previous study showed that in embryonic epithelial cells xMELK is localized not only at the cytokinetic furrow but also at the basolateral cell cortex. The basolateral localization appears to be independent of the cell cycle and developmental stages. The subpopulation of xMELK present in the cell cortex during mitosis was previously named mitotic MELK (mMELK), and the subpopulation remaining at the cell cortex during interphase was named interphasic MELK (iMELK) (Tassan, 2011). However, iMELK remains poorly characterized. Here, we concentrated our interest on the identification of factors responsible for the differences between mMELK and iMELK. We then focused on the question of how the cells can differentially regulate the two xMELK subpopulations, with a particular emphasis on identification of an xMELK partner involved in spatial and temporal regulations of specific localizations of the two subpopulations of xMELK.

## Results

### Two xMELK subpopulations with different spatio-temporal regulation coexist in Xenopus embryo cells

To extend our knowledge of xMELK in Xenopus embryonic cells, a comparative analysis of its localization was undertaken in external epithelial and internal mesenchyme-like cells at two early developmental stages, the blastula and gastrula. Using indirect immunofluorescence staining and confocal microscopy, we analyzed localization of xMELK in parallel with C-cadherin because it is a basolateral membrane adhesion marker.

**Fig. 1.**
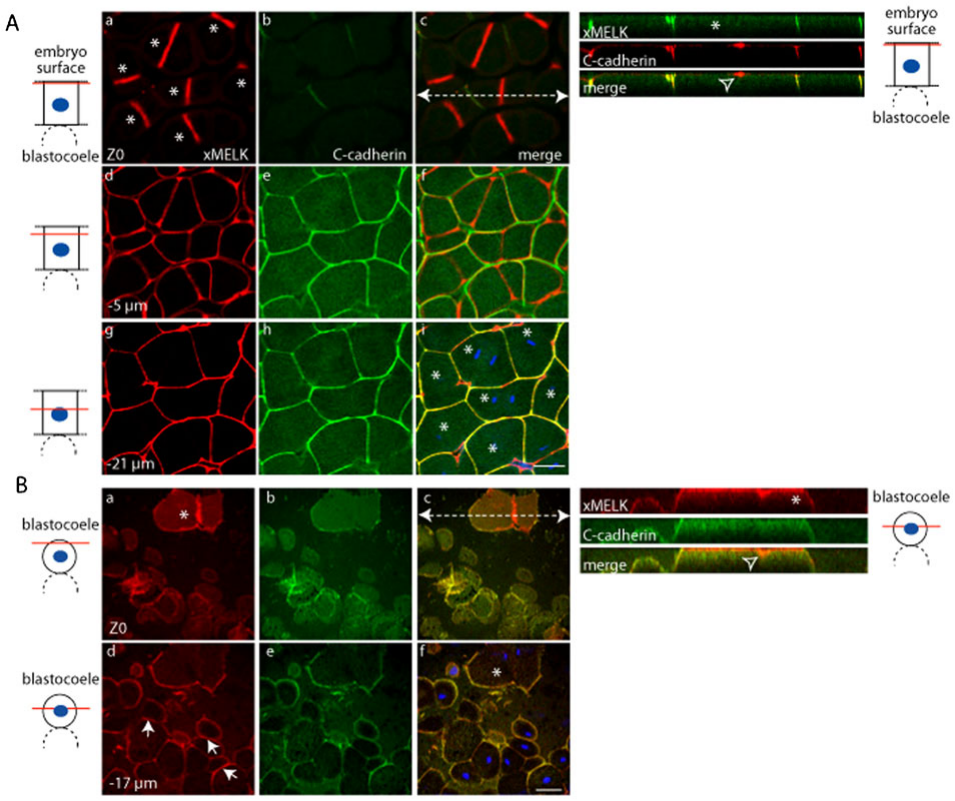
Comparative analysis of xMELK localization in epithelial and mesenchyme-like cells in blastula embryos. Indirect immunofluorescence with anti-xMELK (red) and anti-C-cadherin (green) antibodies was performed on fixed Xenopus albino embryos at blastula stage. (A) Epithelial cells corresponding to the embryonic external cell layer were analyzed by confocal microscopy; 3 optical sections are shown. (B) Internal mesenchyme-like cells facing the blastocoel were observed after dissection of fixed embryos; 2 optical sections are shown. Arrows point to xMELK accumulated at cell–cell contacts. Diagrams on the left: red lines mark the confocal planes relative to embryo surface and blastocoel. Images were merged to visualize co-localization of xMELK with C-cadherin (merge, panels Ac,f,i and Bc,f), DNA is shown (blue). Asterisks indicate cytokinetic cells. White dashed arrows in panels Ac and Bc symbolize the planes used for orthogonal projections of confocal planes shown on the right. Scale bars: 100 µm.

In Xenopus blastula, following the midblastula transition (MBT), the cell division cycles are no longer synchronous. As a consequence both mitotic and interphasic cells are present simultaneously in the post MBT blastula. As previously shown, in the dividing epithelial cells of these blastulae, xMELK accumulates within an equatorial band at the cell surface which ultimately corresponds to the division furrow (Le Page et al., 2011) (Fig. 1Aa,d, asterisks and open arrowhead in orthogonal projections). As expected, C-cadherin is localized at the cell basolateral membrane, but in contrast to xMELK, it does not accumulate within the equatorial band of dividing cells (Fig. 1Ab,e and orthogonal projections). However, C-cadherin and xMELK co-localize at the lateral cell cortex of dividing as well as interphase blastomeres (Fig. 1Ag–i). Internal mesenchyme-like cells situated underneath epithelial cells are more rounded and loose than epithelial cells. In mesenchyme-like cells, similarly to epithelial cells, the xMELK accumulates at the division furrow during cytokinesis (Fig. 1Ba, open arrowhead in orthogonal projections) and during interphase this protein is localized at the cell–cell contacts marked by the presence of C-cadherin (arrows in Fig. 1Bd).

**Fig. 2.**
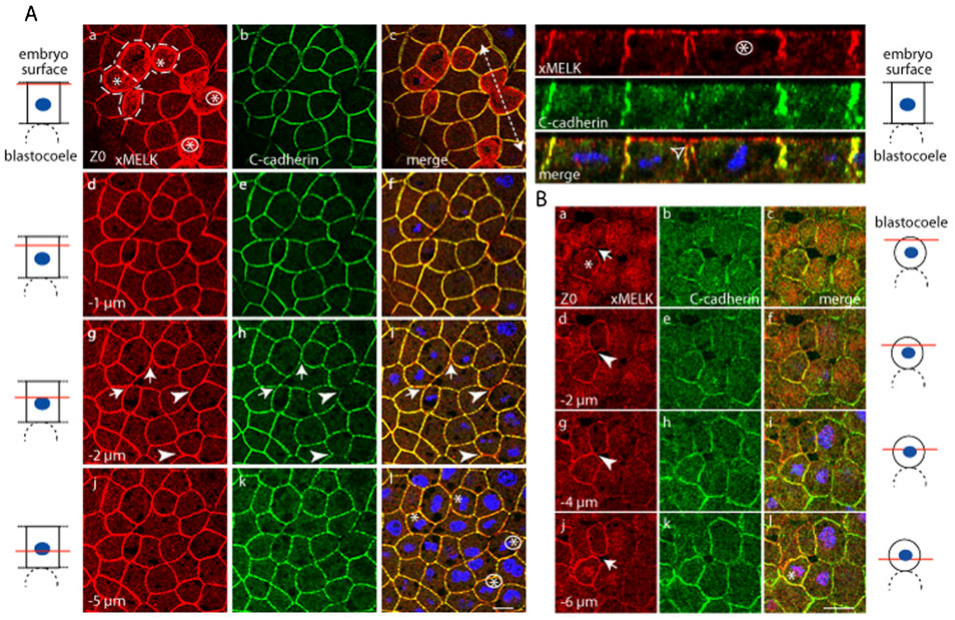
In epithelial cells of gastrula embryos the xMELK and C-cadherin co-localize at the lateral cortex during both interphase and mitosis with the exception of the tip of the ingressing membrane during cytokinesis. (A) Epithelial cells. xMELK (red) and C-cadherin (green) were detected by indirect immunofluorescence with specific antibodies in fixed albino embryos. Diagrams on the left: red lines mark the confocal planes relative to embryo surface. Asterisks indicate cytokinetic cells. Two cells indicated by filled arrow heads (g,h,i) have not yet completed their cytokinesis and two cells indicated by arrows are more advance in their division (their limits are encircled by dashed lines in panel a). Images were merged to visualize co-localization of xMELK with C-cadherin (merge, c,f,i,l), DNA is blue. White dashed arrow in panel c symbolizes the plane used for orthogonal projection of confocal planes shown on the right. The empty arrowhead points to a portion of the ingressing membrane labelled with xMELK antibodies but not with C-cadherin antibodies. (B) As in panel A except that internal, mesenchyme-like cells were analyzed. Diagrams on the right: red lines mark the confocal planes relative to blastocoel. Asterisk indicates a cytokinetic cell. Arrows indicate two daughter cells separated at these confocal planes and arrowheads point to the cell–cell contacts between the two daughter cells. Scale bars: 20 µm.

In gastrula stage embryos, xMELK is no longer present at the equatorial cortex of dividing cells (Le Page et al., 2011). However, it is concentrated along cell periphery and notably marks cytokinetic cells (Fig. 2Aa,c and orthogonal projections; Fig. 2B). In the epithelial cells of gastrula, the cytokinetic furrow ingresses asymmetrically, progressing from the basolateral membrane towards the apical membrane (Le Page et al., 2011). Accordingly, the apical membranes of the cytokinetic cells (marked with encircled asterisks in Fig. 2Aa) are already divided basolaterally (Fig. 2Ag–i, arrowheads) whereas their apical membranes remain unseparated. Interestingly, at the division site, the ingressing cell membrane shows a continuous xMELK labelling whereas the distribution of C-cadherin is weaker and appears as dots like at the cell periphery or sometimes is even absent (Fig. 2Ag–i, arrowheads). This localization was observed for all cytokinetic cells (additional dividing cells are shown in supplementary material Fig. S1). A basolaterally situated gap between plasma membrane of daughter cells is usually observed at this stage of division. In cells advanced in cytokinesis xMELK and C-cadherin show similar distribution along the cell membrane at all confocal planes except at the apical membrane (Fig. 2Aa, cells marked by asterisks). These results suggest that in cytokinetic cells the xMELK is localized along the newly formed plasma membrane between daughter cells including the tip of the ingressing cell membrane. Interestingly, this tip is devoid of C-cadherin (Fig. 2A, orthogonal projections). In contrast to the mitotic cells, in the interphase cells the xMELK is not concentrated at the apical membrane, but is localized at the cell periphery and perfectly co-localizes with C-cadherin at all confocal planes (Fig. 2A, orthogonal projections). High levels of xMELK and C-cadherin are detected throughout the cytoplasm of mesenchyme-like cells. However, in dividing mesenchyme-like cells xMELK consistently accumulates along the cell periphery (cells marked by asterisks in Fig. 2B; supplementary material Fig. S2). Notably, in these cells, furrowing appears symmetric. Like in epithelial cells, in interphase mesenchyme-like cells, xMELK accumulates at cell–cell contacts marked by C-cadherin (Fig. 2B; supplementary material Fig. S3).

Taken together, these results corroborate the previously introduced notion that two xMELK populations harbouring distinct localization behaviours exist in Xenopus embryos, the mitotic xMELK (mMELK) and interphase xMELK (iMELK) (Tassan, 2011). Indeed, during mitosis, mMELK undergoes redistribution to the cell cortex in both epithelial and mesenchyme-like cells indicating that this redistribution is not related to cell polarity. During interphase, iMELK co-localizes with C-cadherin at the cell–cell contacts in both cell types. Our comparative analysis of xMELK localization in epithelial and mesenchyme-like cells shows that iMELK accumulates at cell–cell contacts in both cell types, but it is also concentrated at the apical tip of the lateral membrane of epithelial cells, which seems to be related to their polar organization.

### iMELK is localized at the cell–cell contacts and is highly concentrated at the tight junction

**Fig. 3.**
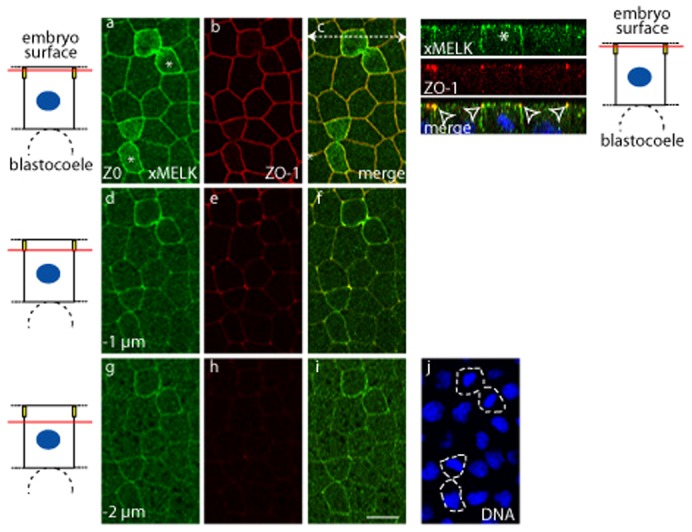
In epithelial cells xMELK accumulates at the tight junctions. Confocal microscopy of indirect immunofluorescence with anti-xMELK (green) and anti-ZO-1 (red) antibodies of epithelial cells from fixed albino embryos at gastrula stage. Three single optical sections spaced by 1 µm are shown. Asterisks indicate cytokinetic cells. Diagrams on the left: red lines mark the confocal planes relative to embryo surface and yellow rectangles symbolize tight junctions. Images were merged to visualize co-localization of xMELK with ZO-1 (merge, c,f,i). DNA is blue (j), dividing cells are indicated by dashed lines. White dashed arrow in panel c symbolizes the plane used for orthogonal projection of confocal planes shown on the right. Arrowheads point to the xMELK which co-localizes with ZO-1 at the tight junctions. Scale bar: 20 µm.

To better characterize iMELK localization in the gastrula epithelium, we compared xMELK localization with that of ZO-1, a component of tight junctions in epithelial cells (in this experiment the anti-xMELK antibody was used more diluted; see Materials and Methods). As shown above, the mMELK is present at the apical surface of cytokinetic cells (Fig. 3, marked by asterisks) and iMELK at the lateral cortex in interphase cells. The fluorescence of iMELK signal decreases rapidly from apical to basal confocal planes but the signal, although faint, persists basally (compare Fig. 3a,d and Fig. 3g). This is especially clear on orthogonal projections in which iMELK appears as a bright fluorescent dot with labelling extending slightly below. As expected for a constituent of the tight junctions, ZO-1 is concentrated at the apical edge of both mitotic and interphase epithelial cells and therefore on orthogonal projections, appears almost exclusively concentrated in dots (Fig. 3b,e and Fig. 3h). Interestingly, iMELK dots perfectly co-localize with ZO-1 (open arrowheads in orthogonal projections) indicating that iMELK is concentrated at the tight junction.

### mMELK relocalization is cell–cell contacts independent in both blastula and gastrula embryos, whereas iMELK localization is dependent on cell–cell contacts in gastrula, but not in blastula

**Fig. 4.**
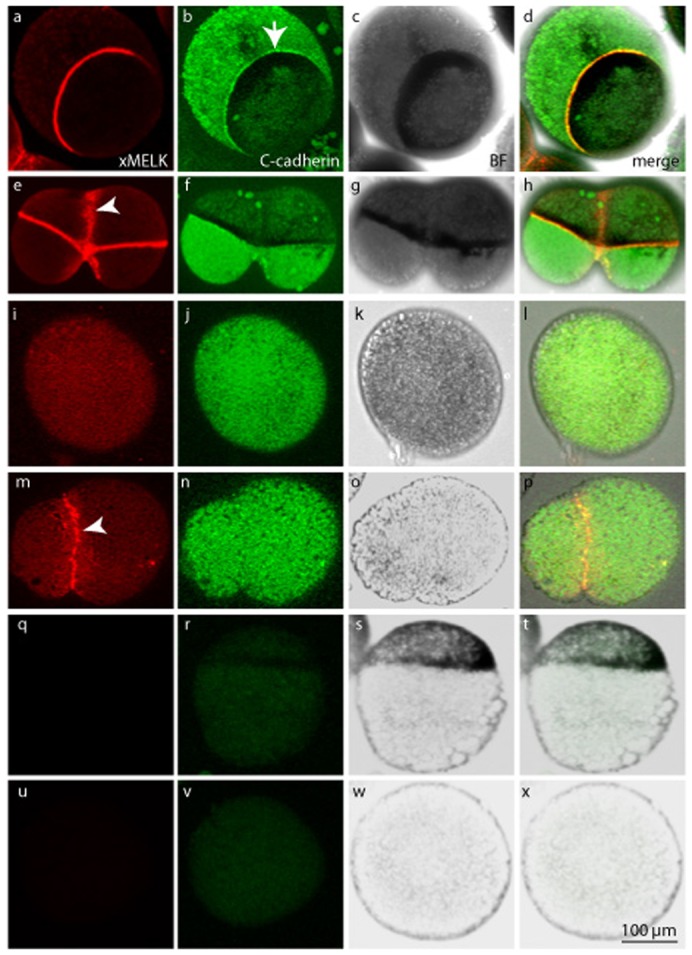
xMELK localization in interphase and dividing cells of dissociated blastula embryos. Pigmented embryos were dissociated by incubation in calcium and magnesium devoid medium. Isolated cells were fixed and immunofluorescence was performed with anti-xMELK (red, a,e,i,m) and anti-C-cadherin (green, b,f,j,n) antibodies. Control cells (q–x) were processed for indirect immunofluorescence like others except that the primary antibody was omitted. Bright field microscopy (BF, grey, c,g,k,o,s,w) shows pigmented epithelial cells (a–h,q–t) and mesenchyme-like cells devoid of pigments (i–p,u–x). Images were obtained by projection of 10 single confocal sections. Images were merged to visualize co-localization of xMELK with C-cadherin (merge, d,h,l,p,t,x). Interphase (a–d,i–l,q–x) and dividing (e–h,m–p) cells are shown. Scale bar: 100 µm.

To test the hypothesis that iMELK localization at the cell periphery correlates with the presence of cell–cell interactions, the contacts between cells were disrupted by dissociating the embryos. First, embryos were incubated in medium deprived of calcium and magnesium ions from the two-cell stage until untreated embryos reached stage 7. In these conditions, embryonic cells lose their contacts and isolated cells can be recovered. Epithelial cells keep their apical–basal polarity after dissociation (Müller and Hausen, 1995) and can be easily discriminated from other cell types by higher content of pigments concentrated in the apical hemisphere. In these cells, C-cadherin is detected at the baso-lateral membrane (the hemisphere devoid of pigment) and is also more concentrated at the border with the apical area in which pigment is concentrated (Fig. 4b, compare with control embryos shown in Fig. 4q–t where only the secondary antibodies were used). However, xMELK is almost exclusively concentrated within a narrow ring which co-localizes with C-cadherin. In cytokinetic epithelial cells, xMELK is detected both as a ring below pigment and as a larger and diffuse band which corresponds to the cytokinetic furrow (Fig. 4e). In the isolated cells, similarly to the cells in intact embryo, C-cadherin is not concentrated at the division site. In mesenchyme-like cells, which are not pigmented, both xMELK and C-cadherin show a disperse distribution (Fig. 4i,j, for the specificity of localization compare with negative control embryos shown in Fig. 4u,x). During cytokinesis, xMELK but not C-cadherin become exclusively detected in a large and diffuse equatorial band (Fig. 4m,n).

**Fig. 5.**
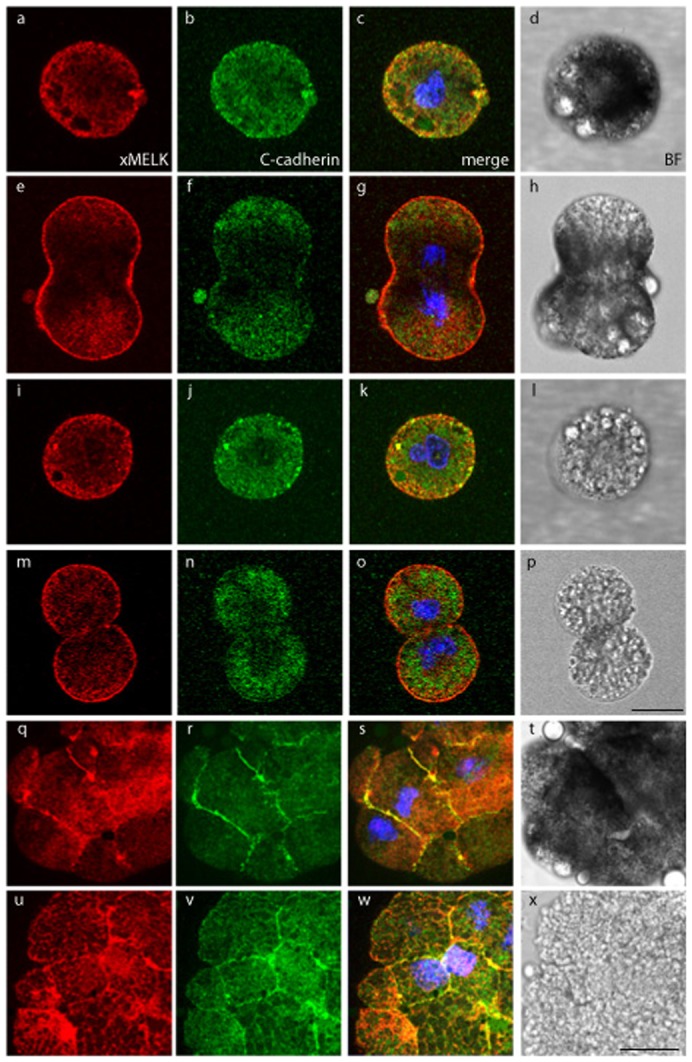
Two xMELK subpopulations have distinct requirement for cell–cell contacts for their localization at the cell cortex. Animal caps of pigmented embryos were dissected and cells were dissociated with trypsin treatment. Cells were left isolated (a–p) or sorted according to their pigmentation and allowed to re-associate for 3 hours (q–x). Cells were fixed, processed for indirect immunofluorescence with anti-xMELK (red) anti-C-cadherin (green) antibodies and observed by confocal microscopy. Single optical sections are shown. Bright field (BF, grey) show pigmented epithelial cells (a–h,q–t) and mesenchyme-like cells devoid of pigment (i–p,u–x). Images were merged together with images of DNA (blue) at the same confocal plane to visualize co-localization of xMELK with C-cadherin (merge, c,g,k,o,s,w). Scale bars: 20 µm.

Because we have previously shown that xMELK localization depends of the developmental stage, we also analyzed xMELK localization in dissociated cells of post-MBT embryos. At this developmental stage, cell cohesion in the epithelium appears too strong to be disrupted by divalent ions deprivation. Therefore, animal cap explants of blastula embryos were manually dissected and dissociated by trypsin treatment. Dissociated epithelial (pigmented, Fig. 5d,h,t) and mesenchyme-like (devoid of pigment, Fig. 5l,p,x) cells were sorted manually using a micropipette and either cultured at low density on an agarose layer to avoid cell–cell re-adhesion or placed into agarose wells to favour cell–cell re-adhesion. We found that in dissociated epithelial cells the distribution of the pigment is no longer asymmetrical contrary to isolated blastula epithelial cells, which suggests that the cells lost their apical–basal polarity. Confocal microscopy of epithelia and mesenchymal-like isolated cells shows that during interphase, the C-cadherin and xMELK are evenly distributed (Fig. 5a–d and Fig. 5i–l, respectively) in contrast to their cortical distribution in cells of the intact embryo. This result indicates that xMELK as well as C-cadherin do not concentrate at the cortex if the cell–cell contacts are disrupted. Interestingly, in both epithelial and mesenchyme-like cytokinetic cells, the xMELK, but not C-cadherin is localized at the cell periphery (Fig. 5). This indicates that the cortical localization of xMELK during cytokinesis is independent of cell–cell contacts. In cells that were allowed to re-aggregate, new cell–cell contacts formed (as indicated by the focused distribution of C-cadherin) and xMELK was found concentrated at the newly formed cell–cell contacts. Altogether, these results indicate that iMELK and mMELK not only differ in their spatio-temporal localization, but, also, in their requirement for the presence of cell–cell contacts (Fig. 5q–t and Fig. 5u–x, respectively). Altogether, our results show that iMELK localization is dependent on cell–cell contacts and that mMELK relocalizes at the cell cortex during cytokinesis independently of cell–cell contacts.

### RACK1 is an xMELK partner

**Fig. 6.**
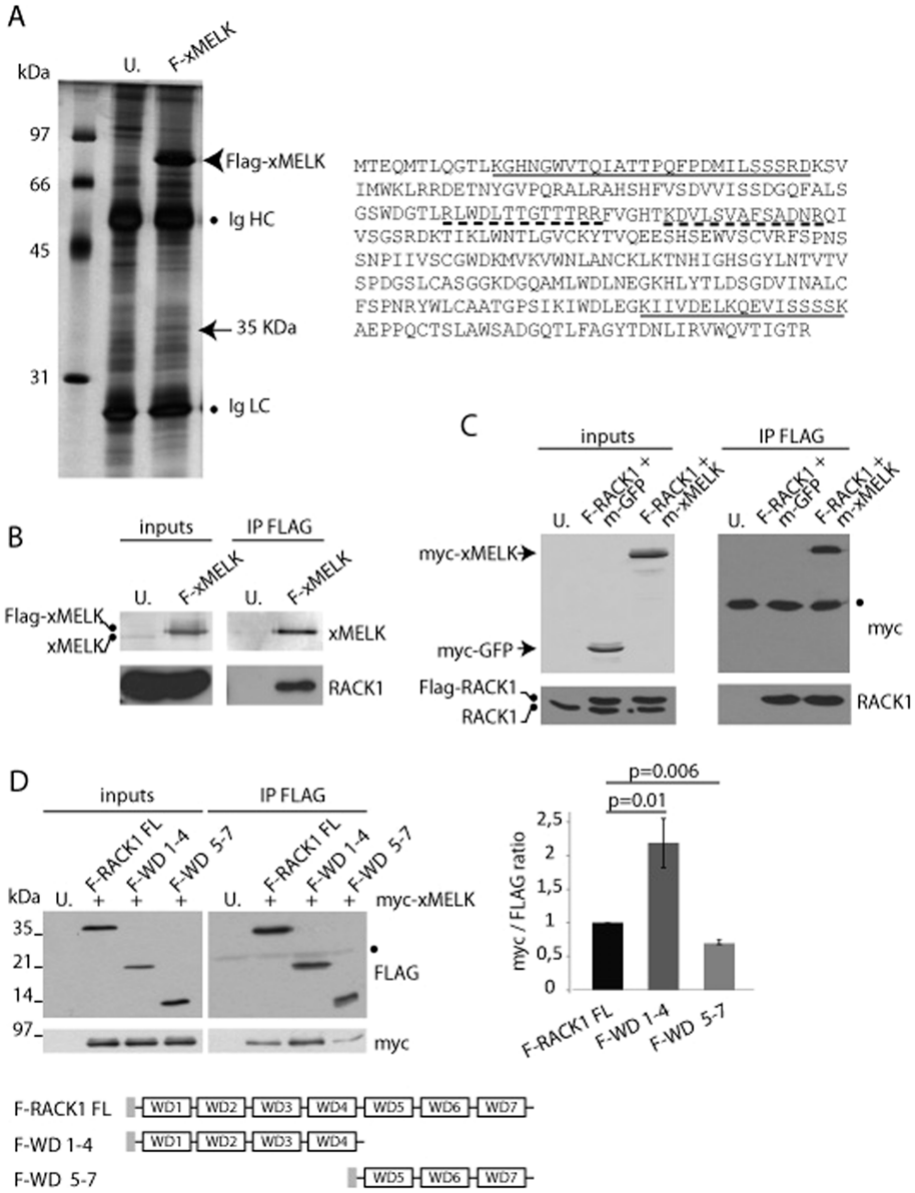
xMELK and RACK1 are in the same complex. (A) Identification of RACK1 as a potential xMELK partner. Proteins extracted from FLAG-xMELK expressing or uninjected control (U.) embryos were immunoprecipitated with anti-FLAG antibodies, separated by SDS-PAGE and silver stained. The 35 kDa band present in the FLAG-xMELK but not in the control immunoprecipitate was cut out from the gel and analyzed by mass spectrometry. Two peptides matching RACK1 protein sequence (underlined) were identified. Two additional peptides were identified in an independent experiment (dashed underline). Ig HC and Ig LC: immunoglobulins heavy and light chains, respectively. (B,C) Validation of xMELK and RACK1 interaction. (B) Proteins were extracted from FLAG-xMELK (F-MELK) expressing or uninjected (U.) embryos (inputs). Proteins were immunoprecipitated with anti-FLAG antibodies (IP FLAG) and Western blots were incubated with anti-xMELK and anti-RACK1 antibodies. (C) Protein extracts (inputs) were prepared from embryos co-expressing FLAG-RACK1 (F-RACK1) and myc-GFP (m-GFP), FLAG-RACK1 and myc-xMELK (m-MELK) or uninjected control embryos. Proteins were immunoprecipitated with anti-FLAG antibodies (IP FLAG) and Western blots were incubated with anti-myc and anti-RACK1 antibodies. (D) xMELK preferentially associates with RACK1 N-terminal domain. Protein extracts (inputs) were prepared from embryos co-expressing myc-xMELK with full length FLAG-RACK1 (F-RACK1 FL), FLAG-RACK1 WD1–4 (F-WD1–4), and FLAG-RACK1 WD5–7 (F-WD5–7) or uninjected (U.) embryos. Proteins were immunoprecipitated with anti-Flag antibodies (IP FLAG) and Western blots were incubated with anti-FLAG and anti-myc antibodies. The histogram on the right represents quantifications of the myc signal obtained in 3 independent immunoprecipitation experiments normalized with the corresponding FLAG signals (myc/FLAG ratio). Error bars denote s.e.m., a *t*-test was performed and p values are indicated above bars. Schematic representation of the RACK1 constructs is shown. The grey box indicates the FLAG tag.

The accumulation of xMELK at the apical junctional complex and the fact that its localization depends on cell–cell contacts suggested that it should interact with putative partners localized at the cell–cell contacts. To test this hypothesis we sought to identify such putative xMELK partner(s). To this end, a synthetic mRNA encoding FLAG-tagged xMELK (FLAG-xMELK) was microinjected into two-cell stage embryos, which were allowed to develop until the gastrula stage. Proteins were immunoprecipitated with anti-FLAG antibodies, separated by SDS-PAGE and analyzed by mass spectrometry. One protein with a molecular weight of 35 kDa was specifically and reproducibly co-immunoprecipitated with FLAG-xMELK, but was not immunoprecipitated from uninjected embryos (Fig. 6A). This protein was submitted to mass-spectrometry analysis. This allowed identification of two peptides matching with the RACK1 (Receptor for Activated Protein Kinase C 1) amino acid sequence (solid lines in Fig. 6A). In the second set of experiments, proteins precipitated with the anti-FLAG antibodies were eluted by the FLAG peptide and directly submitted to mass-spectrometry analysis without prior separation by SDS-PAGE. This allowed identification of two further peptides matching with the RACK1 sequence (dashed lines in Fig. 6A). These results suggested that xMELK and RACK1 are indeed present in the same complex. Because RACK1 is an adaptor molecule that has been previously shown to localize at cell–cell contacts to promote cell–cell adhesion (Mourton et al., 2001) and to regulate membrane localization of diverse partners (Adams et al., 2011), we further investigated the xMELK and RACK1 relationship. To validate mass spectrometry results we used co-immunoprecipitation method. Proteins from FLAG-xMELK expressing embryos were immunoprecipitated using anti-FLAG antibodies and uninjected embryos were used as controls. Precipitated proteins were then analyzed by Western blots using anti-xMELK and anti-RACK1 specific antibodies. Anti-FLAG antibodies immunoprecipitated FLAG-xMELK and a substantial amount of endogenous RACK1 (Fig. 6B). This result shows that RACK1 is specifically present in the FLAG-xMELK immunoprecipitate. To confirm this result, an *in vitro* transcribed mRNA coding FLAG tagged RACK1 (FLAG-RACK1) was co-injected together with myc-tagged xMELK (myc-xMELK) or myc-tagged GFP (Green Fluorescent Protein, m-GFP) mRNAs to Xenopus embryos. Immunoprecipitations were performed using anti-FLAG antibodies and proteins were analyzed by Western blots with anti-FLAG or anti-myc antibodies. FLAG-RACK1 but not the endogenous RACK1 was detected in FLAG precipitates using anti-FLAG antibodies showing that FLAG-RACK1 are co-precipitated (Fig. 6C). Anti-myc antibodies detected myc-xMELK in the FLAG immunoprecipitate but not myc-GFP demonstrating that myc-xMELK is specifically co-immunoprecipitated with FLAG-RACK1. RACK1 consists of the repetition of 7 WD40 domains (scheme in Fig. 6D), each repeat potentially constituting an interaction domain for RACK1 partners. To test if xMELK preferentially interacts with N or C terminal WD40 RACK1 domains, the interaction of myc-xMELK with two FLAG-RACK1 truncated constructs was compared with full length FLAG-RACK1 (FLAG-RACK1 FL). Embryos were co-injected with mRNAs coding for myc-xMELK and FLAG-RACK1 FL or FLAG-RACK1 WD1–4 (in which WD40 domains 5 to 7 have been deleted) or FLAG-RACK1 WD5–7 (in which WD40 domains 1 to 4 have been deleted), FLAG-tagged protein were immunoprecipitated with anti-FLAG antibodies and analyzed by Western blots with anti-FLAG and anti-myc antibodies. As shown in Fig. 6D, myc-xMELK co-immunoprecipitated with the 3 FLAG-RACK1 constructs, but with different affinities. Substantially more of myc-xMELK co-immunoprecipitated with FLAG-RACK1 WD1–4 (2.1 times), and slightly less with FLAG-RACK1 WD5–7 (0.7 times) when compared to full length FLAG-RACK1. Taken together, our results show that xMELK and RACK1 are present in the same protein complex and that xMELK interacts to different degree with the N and C terminal RACK1 domains; preferentially with the N terminal (WD1–4) and less with the C terminal domain (WD5–7).

### RACK1 and iMELK co-localize with ZO-1 at the tight junction in embryo epithelial cells

**Fig. 7.**
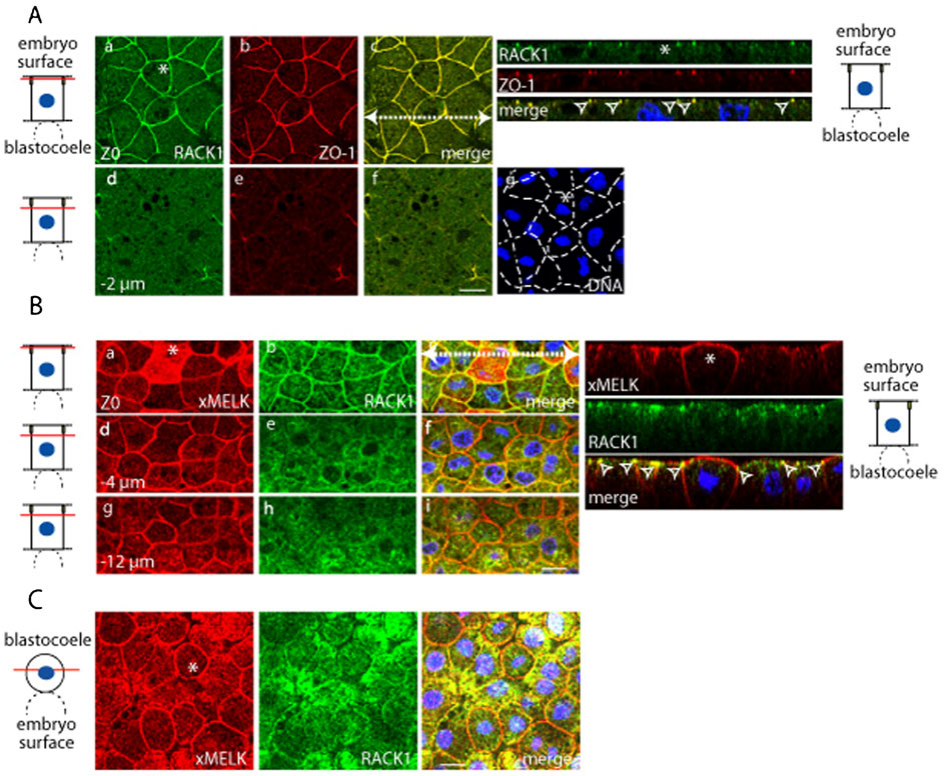
In epithelial cells, RACK1 co-localizes with xMELK at the tight junctions. (A) In gastrula epithelial cells, RACK1 co-localizes with ZO-1 at the tight junctions. Endogenous RACK1 (green, a,d) and ZO-1 (red, b,e) were detected with specific antibodies. Two single optical sections spaced by 2 µm are shown; their positions relative to the embryo surface are symbolized by red lines (diagrams on the left). Yellow rectangles symbolize tight junctions. Images were merged to visualize co-localization of RACK1 with ZO-1 (merge, c,f). DNA is blue (g). Asterisks indicate cytokinetic cell. White dashed arrow in panel c indicates the plane used for orthogonal projection of confocal planes shown on the right. Arrowheads point to the RACK1 which co-localizes with ZO-1 at the tight junctions. (B) RACK1 colocalizes with xMELK at the tight junctions. In epithelial cells, xMELK (green, a,d,g) and RACK1 (red, b,e,h) detected by specific antibodies co-localize at the tight junctions. Diagrams on the left: confocal planes relative to embryo surface are marked by red lines. Yellow rectangles symbolize tight junctions. The asterisk indicates cytokinetic cell. Orthogonal projections of confocal planes are shown on the right. The plane of orthogonal projection is indicated by a white dashed line in panel c. Arrowheads point to the RACK1 which co-localizes with xMELK at the tight junctions. (C) xMELK (red) and RACK1 (green) were detected by indirect immunofluorescence with specific antibodies in mesenchymal-like cells. Confocal plane relative to the blastocoel is indicated by a red line on the diagram on the left. Asterisk indicates cytokinetic cell. Images were merged together with images of DNA (blue) at the same confocal planes to visualize co-localization of xMELK with RACK1 (merge). Scale bars: 20 µm.

Because the results of co-immunoprecipitation indicated that xMELK and RACK1 are present in the same complex, it was important to determine in which cellular compartment these two proteins could potentially interact, and if RACK1 interaction is specific to one of the two xMELK subpopulations. To answer these questions, we examined endogenous RACK1 localization in fixed Xenopus embryos. We show that in the interphase and mitotic epithelial cells the RACK1 localizes at the cell–cell contacts and co-localizes with ZO-1 (Fig. 7A and orthogonal projections). We also compared endogenous xMELK and endogenous RACK1 localizations and found that RACK1 does not re-localize to the cell cortex in cytokinetic cells (Fig. 7Ba–c). This result was further supported by the fact that in living blastula and gastrula embryos (supplementary material Fig. S4A,B, respectively), the GFP-tagged RACK1 does not accumulate at the division furrow or redistribute to the cell cortex during cytokinesis. This suggests that xMELK and RACK1 do not interact at these cellular locations during cytokinesis. In contrast, the two proteins co-localize at the tight junctions (Fig. 7B, open arrowheads in orthogonal projections). Interestingly, in mesenchyme-like cells, RACK1 is diffusely distributed and only low levels are present at the cell cortex during both mitosis and interphase (Fig. 7C). Taken together, these results show that RACK1 does not follow the characteristic relocalization behaviour of mMELK. However, RACK1 localization follows the localization pattern of iMELK suggesting that in the epithelial cells it may specifically interact with iMELK at the tight junctions.

### RACK1 regulates iMELK localization at the cell cortex

**Fig. 8.**
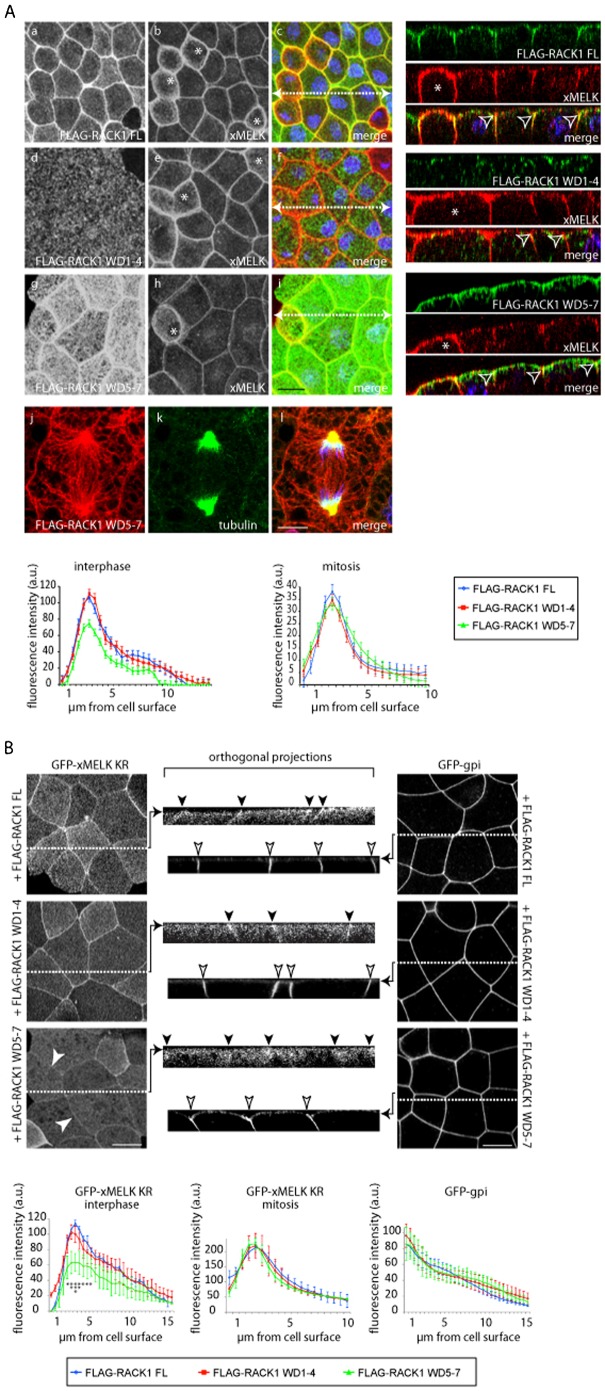
RACK1 regulates localization of iMELK. (A) Gastrula embryos expressing FLAG-RACK1 FL, FLAG-RACK1 WD1–4 and FLAG-RACK1 WD5–7 were fixed and processed for indirect immunofluorescence with anti-FLAG (a,d,g) and anti-xMELK antibodies (b,e,h). Pictures were merged (merge, c,f,i) together with pictures of DNA (blue) at the same confocal planes to visualize co-localization of xMELK (red) with FLAG-RACK1 constructs (green). Embryos expressing FLAG-RACK1 WD5–7 were incubated with a rabbit polyclonal anti-FLAG (j) and a mouse monoclonal anti-tubulin (k) antibody. Pictures were merged together with pictures of DNA (blue) to visualize FLAG-RACK1 WD5–7 and microtubules. White dashed arrows in panels c, f and i indicate the plane used for orthogonal projections of confocal planes shown on the right. Asterisks indicate cytokinetic cells. Arrowheads point on xMELK concentrated at the tight junctions. Scale bars: 20 µm (a–i), 10 µm (j–l). Intensity of the xMELK fluorescent signals at the cell–cell contacts in embryos expressing FLAG-RACK1 FL, FLAG-RACK1 WD1–4 and FLAG-RACK1 WD5–7 were quantified in interphase and mitotic cells for each 0.5 µm confocal plane. (B) Embryos were coinjected with FLAG-RACK1 FL, FLAG-RACK1 WD1–4 and FLAG-RACK1 WD5–7 mRNAs with GFP-xMELK KR or GFP-gpi mRNAs. White dashed lines mark the plane used for orthogonal projections of confocal planes shown in the center. Arrows points to the apical junctional complex. Black and white arrows points to GFP-xMELK and GFP-gpi, respectively. Scale bars: 20 µm. The intensity of the GFP-xMELK KR and GFP-gpi fluorescent signals at the cell–cell contacts in embryos expressing FLAG-RACK1 FL, FLAG-RACK1 WD1–4 and FLAG-RACK1 WD5–7 was quantified in interphase and mitotic cells for each 0.5 µm confocal plane. Statistical analysis was performed. *GFP-xMELK KR+ FLAG-RACK1 WD5–7 is significantly different from GFP-xMELK KR+ FLAG-RACK1 FL at p<0.005, **p<0.002, ***p<0.0003. Note that asterisks are oriented vertically on the figure. For other points 0.5>p>0.005.

To explore if RACK1 could contribute to iMELK localization at the cell cortex, we tried to knockdown RACK1 in embryos using Morpholinos. Although we microinjected increasing amounts of Morpholinos and we harvested embryos at diverse stages of development, the level of endogenous RACK1 was not detectably affected (supplementary material Fig. S5). Thus, this approach failed; however, we showed (Fig. 6) that iMELK preferentially interacts with the RACK1 WD1–4 domains and to a lesser degree with the WD5–7 domains. Therefore, as an alternative approach to RACK1 knockdown, we tested if, and to what extent, the overexpression of these RACK1 truncated constructs could interfere with the localization of endogenous iMELK. FLAG-RACK1 FL, FLAG-RACK1 WD1–4 and FLAG-RACK1 WD5–7 were expressed in embryos and their subcellular localizations were analyzed. Similarly to the endogenous RACK1, the FLAG-RACK1 FL is concentrated at cell–cell contacts in epithelial cells (Fig. 8Aa,c). In contrast, the FLAG-RACK1 WD1–4 is distributed, in the form of dots, throughout the cytoplasm (Fig. 8Ad,f). This result indicates that WD1–4 domain does not localize at the cell–cell contacts. However, FLAG-RACK1 WD5–7 is concentrated at the apical junctional complex of epithelial cells similarly to FLAG-RACK1 FL (Fig. 8Ag,i). In addition, FLAG-RACK1 WD5–7 is also localized within the cytoplasm on the network resembling microtubules (Fig. 8Ag, the large central cell, Fig. 8Aj–l; supplementary material Fig. S6) and is more concentrated at the apical cortex. Taken together, these results suggest that WD5–7 domain regulates RACK1 localization at the junctional complex, and that WD1–4 may be involved in the specificity of RACK1 subcellular localization by restricting its localization to specific structures or areas within the cell (e.g. along the microtubules). To analyze if the expression of truncated RACK1 constructs could influence xMELK localization, the endogenous xMELK localization was detected with specific anti-xMELK antibodies. In FLAG-RACK1 WD1–4 expressing embryos, the xMELK localization appears similar to that in FLAG-RACK1 FL embryos (Fig. 8Ab,e). In contrast, xMELK accumulation at the cell–cell contacts was clearly reduced in FLAG-RACK1 WD5–7 expressing embryos (Fig. 8Ah). Indeed, quantification of the fluorescent signal showed that xMELK cortical localization was about 40% lower in FLAG-RACK1 WD5–7 expressing embryos than in FLAG-RACK1 FL and FLAG-RACK1 WD1–4 expressing embryos (Fig. 8A, histogram on the left side, interphase). Importantly, this diminution was observed for interphase cells but not for mitotic cells (Fig. 8A, histogram on the right side, mitosis), indicating that this effect is specific for iMELK. A similar effect on iMELK localization was observed in living embryos co-expressing GFP-xMELK KR, an inactive xMELK mutant unable to induce cytokinesis defects (Le Page et al., 2011) in combination with FLAG-RACK1 FL, FLAG-RACK1 WD1–4 or FLAG-RACK1 WD5–7 (Fig. 8B). Similar to the endogenous xMELK, the accumulation of GFP-xMELK KR at the cell–cell contacts was substantially reduced when co-expressed with FLAG-RACK1 WD5–7 (Fig. 8B, arrowheads, left). Quantification of the fluorescent signal showed that GFP-xMELK KR was about 40% lower in FLAG-RACK1 WD5–7 than in FLAG-RACK1 FL and FLAG-RACK1 WD1–4 (Fig. 8B, histogram on the left side, GFP-xMELK KR interphase). As for the endogenous protein, this diminution was observed for interphase cells but not for mitotic cells (Fig. 8B, histogram in the middle, GFP-xMELK KR mitosis). This reduction in GFP-xMELK KR level was specific for this protein accumulation at cell cortex because no reduction was observed for the plasma membrane protein marker GFP-gpi (Fig. 8B, histogram on the right side, GFP-gpi). Altogether, these results suggest that the expression of FLAG-RACK1 WD5–7, which localizes to cell–cell contacts, reduces accumulation of iMELK at the cell–cell junctions. Taken together, our results are consistent with a model in which iMELK is localized at the lateral cell cortex and the apical junctional complex where it associates with RACK1.

## Discussion

### Two xMELK subpopulations are differently regulated

According to their spatial and temporal regulations, two xMELK subpopulations can be distinguished in Xenopus embryonic cells. The mitotic xMELK subpopulation, mMELK, shows a highly dynamic subcellular relocalization at the cell cortex specifically during mitosis (Le Page et al., 2011). This localization is subjected to developmental regulation in Xenopus embryos. In the early embryos, shortly before the onset of cytokinesis, the mMELK concentrates, together with other cytokinetic proteins, in an equatorial band, which ultimately corresponds to the cell division furrow. However, later in development, in the gastrula, the mMELK is no longer concentrated in the equatorial band. Herein, we show that in the dividing cells of gastrula embryos, the mMELK is redistributed to the cell cortex. The cortical localization was observed in both epithelial and mesenchyme-like cells, which suggests that the cortical localization of MELK may be a common feature shared by diverse cell types. This is in agreement with our previous results showing that in the HeLa human cell line, starting from anaphase until mitotic exit, the MELK is also distributed at the cell cortex (Chartrain et al., 2006).

The interphase xMELK subpopulation, iMELK, is localized at the lateral cell cortex independently of the cell-cycle phase and developmental stage (Le Page et al., 2011). In the present study, we show that iMELK has a lateral localization in both epithelial and mesenchyme-like cells and that it is concentrated at the junctional complex of epithelial cells where it co-localizes with the tight junction protein ZO-1. Interestingly, MELK was previously identified in a proteomic analysis of purified tight junction complex from the human epithelial T84 cell line (Tang, 2006). Our results obtained with Xenopus embryonic epithelial cells are in agreement with the xMELK being associated with the tight junction complex.

The two subpopulations of xMELK have distinct requirements for their proper intracellular localizations. Whereas iMELK depends on cell–cell contacts to be localized at the lateral cortex, mMELK localizes independently of cell–cell contacts either at the division site in cells isolated from blastula or along cell periphery in the cells isolated from gastrula embryos. Remarkably, in cells isolated from blastula embryos, which, as shown by localization of pigment and C-cadherin, remain polarized, we observed that iMELK remains concentrated at the lateral cortex. In contrast, in epithelial cells isolated from gastrula embryos, which lost their polarity, as shown by redistribution of pigments and C-cadherin throughout the cell, the xMELK is no longer concentrated at the cell cortex during interphase. This result suggests that iMELK localization correlates with the epithelial cell polarity. Further studies are needed to clarify and characterize the regulation of xMELK by cell polarity events.

We have previously shown that in the epithelial cells of gastrula embryos the cytokinesis furrow progresses asymmetrically from the baso-lateral membrane towards the cell apex (Le Page et al., 2011). Here, we show that mesenchyme-like cells do not have an asymmetric furrowing. This suggests that epithelial cell polarity might play an important role in regulation of asymmetric furrowing. We also observed that in the cytokinetic cells of gastrula embryos, the mMELK is present at the tip of the asymmetric ingressing membrane, whereas C-cadherin is present in discontinuous pattern along the ingressing membrane (Fig. 2). This indicates that mMELK is present at the newly formed membrane between the two daughter cells. In the cytokinetic cells of whole blastula embryos, mMELK was highly concentrated at the equatorial cortex whereas C-cadherin was not. This is consistent with the notion that mMELK localization is cell–cell contact independent.

At present, it is not known how the xMELK becomes localized at the newly formed membrane between daughter cells when they exit mitosis. The mMELK could be either converted or replaced by iMELK. This event may be linked to the formation of newly formed junctional complexes between the two daughter cells. These new junctions would allow recruitment of iMELK. Available information on mechanisms regulating xMELK localization from other species may shed some light on this issue. Our studies on human cultured cells showed that a part of the MELK kinase regulatory domain, (the C-terminal domain, which includes the KA1 domain, Kinase Associated domain 1), is involved in MELK localization at the cell cortex during mitosis (Chartrain et al., 2006). In agreement with our finding, it has been recently shown that in various kinases, including MELK, the KA1 domain is responsible for their association with cellular membranes (Moravcevic et al., 2010). However, the higher concentration of iMELK at the cell–cell contacts in epithelial cells led us to hypothesize that iMELK may interact with a putative partner present in this particular subcellular location.

### Identification of RACK1-xMELK complex

In this study, we identified RACK1 as a new xMELK partner. We show that RACK1 is localized at the cell–cell contacts in Xenopus embryonic mesenchyme-like cells. This result is in agreement with previous reports showing that RACK1 is localized at cell–cell junctions in HT-29 human colon cancer cell line and mink Mv 1 Lu cells (Swaminathan and Cartwright, 2012; Mourton et al., 2001). We also show that in polarized Xenopus embryonic epithelial cells RACK1 co-localizes with ZO-1 at the apical cell–cell junction. Interestingly, RACK1 localization varies depending on the cell type. This is reminiscent of ZO-1 localization. Indeed, in epithelial cells, ZO-1 is concentrated in tight junctions, where it interacts with integral transmembrane proteins including occludin, claudins and JAMs (junctional adhesion molecules), which are specifically enriched at this type of junctions. However, in non-epithelial cells, ZO-1 is localized at cadherin based intercellular junctions where it interacts with alpha-catenin (Itoh et al., 1997). It was already shown that RACK1 associates with several transmembrane receptors such as β1 integrins (Liliental and Chang, 1998; Besson et al., 2002), RPTPmu (Receptor Protein Tyrosine Phosphatase) (Mourton et al., 2001) and PTK7 (Protein Tyrosine kinase) (Wehner et al., 2011). It was also shown that RPTPmu recruits RACK1. However, at present it is unknown how RACK1 is recruited to the tight junction complexes in epithelial cells. Our results obtained from living and fixed embryos show that, during cytokinesis, in contrast to mMELK, RACK1 does not relocalize to the division furrow in blastula or along the cell cortex in gastrula. This absence of co-localization between mMELK and RACK1 indicates that during cytokinesis RACK1 does not associate with mMELK. In contrast, in epithelial cells, we show that xMELK and endogenous RACK1 co-localize at cell–cell contacts and the two of them concentrate together with ZO-1 at the tight junctions. This result indicates that the iMELK localized at the apical junction, but not at the lateral membrane, can interact with RACK1. Therefore, only a part of iMELK may be associated with RACK1. We also show that the RACK1 WD5–7 domain is involved in RACK1 localization at the cell–cell contacts in the embryo epithelium. Though RACK1 WD1–4 does not direct RACK1 to cell–cell contacts, our results suggest that it may regulate its localization by restricting its diffusion within the cell. As shown in fixed and living embryos, the expression of RACK1 WD5–7 inhibits localization of the endogenous xMELK as well as GFP-xMELK KR to the cell–cell contacts. Our data show that RACK1 WD5–7 localizes to the apical cell–cell contacts. Therefore, we hypothesize that this construct could compete with the endogenous RACK1 for its localization at the apical junctional complex. Because RACK1 WD5–7 concomitantly shows reduced association with xMELK, it could exert a negative dominant effect resulting in the decrease in iMELK localization at the apical junctional complex. Importantly, in cytokinetic cells, RACK1 WD5–7 expression does not appear to affect mMELK localization. This result is in agreement with our previous observations indicating that RACK1 does not follow mMELK localization during cytokinesis. Taken together, our results suggest that RACK1 and iMELK specifically interact at the apical junctional complexes. Further studies will be needed to determine if the iMELK subpopulation localized at the lateral membrane can ultimately localize at the tight junction or if the association of iMELK with the lateral membrane is a prerequisite to its association with the apical junctional complex.

We have recently shown that xMELK is involved in cytokinesis in Xenopus embryos (Le Page et al., 2011). Whether iMELK, although not regulated during mitosis, also participates in cytokinesis will need further studies. Our present study reveals that the two xMELK subpopulations, mMELK and iMELK, show not only distinct spatial and temporal regulation but also have distinct cell–cell contact requirements for their subcellular localizations. In addition, they also differ in their ability to form a complex with RACK1.

## Materials and Methods

### Preparation and microinjection of Xenopus embryos

Xenopus laevis albino and wild-type adults were obtained from the Biological Resources Centre (CRB, Rennes, France). Embryos were prepared and microinjected as described previously (Le Page et al., 2011).

### Dissociation of embryos

For the early embryos studies, the isolated cells were obtained as described previously (Müller and Hausen, 1995). Briefly, after the first cell division, embryos were extensively washed in calcium and magnesium free medium (CMFM, 88 mM NaCl, 1 mM KCl, 2.5 mM NaHCO_3_, 5 mM HEPES pH 7.8) and allowed to develop in the same medium. When control embryos (incubated in calcium and magnesium containing medium) reached stage 7 (Nieuwkoop and Faber, 1994), their vitelline envelope was removed manually, and cells were fixed in 2% TCA in CMFM and used for indirect immunofluorescence staining.

To dissociate post-MBT embryos, the animal caps from ten stage 9 blastulae were dissected, washed in CMFM medium and incubated at room temperature in 0.5% trypsin–EDTA solution (GIBCO). Cells were dissociated by gentle pipetting up and down with a siliconized tip. Dissociation was carefully monitored under a stereoscope. After dissociation, cells were washed in MBS medium (88 mM NaCl, 1 mM KCl, 1 mM MgSO_4_, 7 mM CaCl_2_, 2.5 mM NaHCO_3_, 5 mM HEPES pH 7.8). Using a 140-µm diameter micropipette, pigmented (epithelial) cells and cells devoid of pigment (mesenchyme-like) were sorted manually. Both types of cell were cultured separately either on a flat layer of agarose to avoid cell–cell re-adhesion or inside of agarose wells to favour cell–cell re-adhesion. After 3 hours at 21°C, cells in agarose wells have adhered to each other and formed compact aggregates. Both isolated and aggregated cells were fixed in 2% TCA and processed for indirect immunofluorescence staining.

### Plasmids construction and *in vitro* transcription

pT7T-FLAG-xMELK, pT7T-myc-xMELK, pT7T-myc-GFP, pT7T-RACK1 FL, pT7T-RACK1 WD1–4 and pT7T-RACK1 WD5–7 were obtained by PCR amplification of xMELK, GFP and human RACK1 cDNAs, respectively. Primer sequences included sites for restriction enzymes and the FLAG and myc sequences. PCR products were cloned at EcoR V and Spe I sites in pT7T. Constructs were verified by sequencing. *In vitro* transcription was performed with mMessage mMachine transcription kit according to the manufacturer's instructions (Ambion).

### Extraction of proteins, immunoprecipitation and Western blot

Uninjected and FLAG-xMELK injected embryos (50 of each) were homogenized in EB buffer (10 mM Hepes, pH 7.7, 100 mM KCl, 2 mM MgCl_2_, 5 mM, EGTA, 5 mM DTT, 1% IGEPAL CA-630, 5% glycerol) supplemented with pepstatine, leupeptine, chymostatin, PMSF at 10 µM each, 40 mM NaF, 40 mM β-glycerophosphate, and 2.5 µM okadaic acid. Extracts were clarified by centrifugation at 14,000*g* for 15 min at 4°C. Supernatants containing proteins were incubated for 1 h at 4°C with anti-FLAG immunoglobulins pre-adsorbed on protein A magnetic beads (Dynabeads, Invitrogen). Beads were then extensively washed with EB buffer and eluted proteins were boiled in sample buffer. Alternatively, elution was performed with 5 mg/ml FLAG peptide (Sigma) and eluted proteins were boiled in sample buffer.

Western blots were performed as described previously (Le Page et al., 2011). Following antibodies were used: affinity purified anti-xMELK (L2, 0.2 µg/ml) (Blot et al., 2002), anti-RACK1 (BD Transduction Laboratories, 1:1000), monoclonal anti-FLAG (M2, Sigma, 0.5 µg/ml) and monoclonal anti-myc (clone 9E10, 1:10). Secondary anti-rabbit and anti-mouse peroxidase-coupled antibodies were from Jackson.

### Mass spectrometry

Protein digestion was performed as described previously (Shevchenko et al., 1996) and peptides were desalted and concentrated using miniaturized micro-extraction tips (Rappsilber et al., 2003). Subsequently, tryptic peptides were analyzed by nanoLC-MS/MS using a nanoACQUITY ultra performance liquid chromatography system (Waters, UK) coupled to a LTQ-Orbitrap (Thermo, Germany) mass spectrometer. Samples were injected onto a silica reversed-phase capillary column (New Objective, USA) packed with 3-µm ReproSil-Pur C18-AQ (Dr Maisch GmbH, Germany). Peptides were separated by a stepwise 75-min gradient of 0–100% between buffer A (0.2% formic acid in water) and buffer B (0.2% formic acid in acetonitrile) at a flow rate of 200 nL/min. The mass spectrometer was operated in data dependent MS/MS mode to automatically switch between MS survey and MS/MS fragmentation scans of the five most abundant precursor ions. Peak lists were generated using DTA supercharge (Schulze and Mann, 2004) and searched using the Mascot (Matrix Science, UK) software package with carbamidomethyl cysteine as a fixed modification and oxidized methionine and phosphorylation as variable modifications. Searches were performed with a precursor mass tolerance of 5 ppm and fragment ion tolerance of 0.7 Dalton.

### Indirect immunofluorescence

Whole embryos and dissected animal caps were fixed with TCA and treated for indirect immunofluorescence as described previously (Le Page et al., 2011). The following antibodies were used: affinity-purified anti-xMELK (Blot et al., 2002) (final concentration 1 µg/ml, except in Fig. 3, final concentration 300 ng/ml); anti-C-cadherin (clone 6B6 Developmental Studies Hybridoma Bank, 1:200); anti-RACK1 (BD Transduction Laboratories, 1:500), anti-ZO-1 (Zymed, 1:200), anti-tubulin (TUB 2.1, Sigma, 1:200), anti-FLAG (mouse monoclonal, M2, Sigma, 1:500) and anti-FLAG (rabbit polyclonal, Sigma, 1:200). Secondary antibodies were anti-rabbit-alexa-488 or anti-mouse-alexa-555 (Molecular Probes, 1:200). DNA was stained with TO-PRO-3 (Invitrogen, 0.5 µg/ml). Fixed embryos were mounted in Vectashield (Vector) for observations.

### Imaging

Imaging was performed using a Leica SP5 confocal microscope with a 40× HC Plan-APO- ON 1.25 and 63× HCX Plan-APO- ON 1.4 oil immersion objective lens (Microscopy platform, Biosit) and the ImageJ software (Rasband, W.S., http://rsb.info.nih.gov/ij). Figures were assembled in Adobe Photoshop and Adobe Illustrator (Adobe Systems, Inc.).

## Supplementary Material

Supplementary Material

## References

[b1] Adams D. R. Ron D. Kiely P. A. (2011). RACK1, A multifaceted scaffolding protein: Structure and function. Cell Commun. Signal. 9, 22 10.1186/1478-811X-9-22 21978545 PMC3195729

[b2] Badouel C. Körner R. Frank-Vaillant M. Couturier A. Nigg E. A. Tassan J. P. (2006). M-phase MELK activity is regulated by MPF and MAPK. Cell Cycle 5, 883–889 10.4161/cc.5.8.2683 16628004

[b3] Badouel C. Chartrain I. Blot J. Tassan J. P. (2010). Maternal embryonic leucine zipper kinase is stabilized in mitosis by phosphorylation and is partially degraded upon mitotic exit. Exp. Cell Res. 316, 2166–2173 10.1016/j.yexcr.2010.04.019 20420823

[b4] Besson A. Wilson T. L. Yong V. W. (2002). The anchoring protein RACK1 links protein kinase Cepsilon to integrin beta chains. Requirements for adhesion and motility. J. Biol. Chem. 277, 22073–22084 10.1074/jbc.M111644200 11934885

[b5] Blot J. Chartrain I. Roghi C. Philippe M. Tassan J.-P. (2002). Cell cycle regulation of pEg3, a new Xenopus protein kinase of the KIN1/PAR-1/MARK family. Dev. Biol. 241, 327–338 10.1006/dbio.2001.0525 11784115

[b6] Chartrain I. Couturier A. Tassan J. P. (2006). Cell-cycle-dependent cortical localization of pEg3 protein kinase in Xenopus and human cells. Biol. Cell 98, 253–263 10.1042/BC20050041 16159311

[b7] Cordes S. Frank C. A. Garriga G. (2006). The C. elegans MELK ortholog PIG-1 regulates cell size asymmetry and daughter cell fate in asymmetric neuroblast divisions. Development 133, 2747–2756 10.1242/dev.02447 16774992

[b8] Davezac N. Baldin V. Blot J. Ducommun B. Tassan J. P. (2002). Human pEg3 kinase associates with and phosphorylates CDC25B phosphatase: a potential role for pEg3 in cell cycle regulation. Oncogene 21, 7630–7641 10.1038/sj.onc.1205870 12400006

[b9] Gray D. Jubb A. M. Hogue D. Dowd P. Kljavin N. Yi S. Bai W. Frantz G. Zhang Z. Koeppen H. (2005). Maternal embryonic leucine zipper kinase/murine protein serine-threonine kinase 38 is a promising therapeutic target for multiple cancers. Cancer Res. 65, 9751–9761 10.1158/0008-5472.CAN-04-4531 16266996

[b10] Heyer B. S. Warsowe J. Solter D. Knowles B. B. Ackerman S. L. (1997). New member of the Snf1/AMPK kinase family, Melk, is expressed in the mouse egg and preimplantation embryo. Mol. Reprod. Dev. 47, 148–156 10.1002/(SICI)1098-2795(199706)47:2<148::AID-MRD4>3.0.CO;2-M 9136115

[b11] Itoh M. Nagafuchi A. Moroi S. Tsukita S. (1997). Involvement of ZO-1 in cadherin-based cell adhesion through its direct binding to alpha catenin and actin filaments. J. Cell Biol. 138, 181–192 10.1083/jcb.138.1.181 9214391 PMC2139940

[b12] Jung H. Seong H. A. Ha H. (2008). Murine protein serine/threonine kinase 38 activates apoptosis signal-regulating kinase 1 via Thr 838 phosphorylation. J. Biol. Chem. 283, 34541–34553 10.1074/jbc.M807219200 18948261 PMC3259894

[b13] Le Page Y. Chartrain I. Badouel C. Tassan J. P. (2011). A functional analysis of MELK in cell division reveals a transition in the mode of cytokinesis during Xenopus development. J. Cell Sci. 124, 958–968 10.1242/jcs.069567 21378312

[b14] Liliental J. Chang D. D. (1998). Rack1, a receptor for activated protein kinase C, interacts with integrin beta subunit. J. Biol. Chem. 273, 2379–2383 10.1074/jbc.273.4.2379 9442085

[b15] Lin M. L. Park J. H. Nishidate T. Nakamura Y. Katagiri T. (2007). Involvement of maternal embryonic leucine zipper kinase (MELK) in mammary carcinogenesis through interaction with Bcl-G, a pro-apoptotic member of the Bcl-2 family. Breast Cancer Res. 9, R17 10.1186/bcr1650 17280616 PMC1851384

[b16] Marie S. K. Okamoto O. K. Uno M. Hasegawa A. P. Oba-Shinjo S. M. Cohen T. Camargo A. A. Kosoy A. Carlotti C. G. Jr Toledo S. (2008). Maternal embryonic leucine zipper kinase transcript abundance correlates with malignancy grade in human astrocytomas. Int. J. Cancer 122, 807–815 10.1002/ijc.23189 17960622

[b17] Moravcevic K. Mendrola J. M. Schmitz K. R. Wang Y. H. Slochower D. Janmey P. A. Lemmon M. A. (2010). Kinase associated-1 domains drive MARK/PAR1 kinases to membrane targets by binding acidic phospholipids. Cell 143, 966–977 10.1016/j.cell.2010.11.028 21145462 PMC3031122

[b18] Mourton T. Hellberg C. B. Burden-Gulley S. M. Hinman J. Rhee A. Brady-Kalnay S. M. (2001). The PTPmu protein-tyrosine phosphatase binds and recruits the scaffolding protein RACK1 to cell-cell contacts. J. Biol. Chem. 276, 14896–14901 10.1074/jbc.M010823200 11278757

[b19] Müller H. A. Hausen P. (1995). Epithelial cell polarity in early Xenopus development. Dev. Dyn. 202, 405–420 10.1002/aja.1002020410 7626797

[b20] Nakano I. Paucar A. A. Bajpai R. Dougherty J. D. Zewail A. Kelly T. K. Kim K. J. Ou J. Groszer M. Imura T. (2005). Maternal embryonic leucine zipper kinase (MELK) regulates multipotent neural progenitor proliferation. J. Cell Biol. 170, 413–427 10.1083/jcb.200412115 16061694 PMC2171475

[b21] Nakano I. Masterman-Smith M. Saigusa K. Paucar A. A. Horvath S. Shoemaker L. Watanabe M. Negro A. Bajpai R. Howes A. (2008). Maternal embryonic leucine zipper kinase is a key regulator of the proliferation of malignant brain tumors, including brain tumor stem cells. J. Neurosci. Res. 86, 48–60 10.1002/jnr.21471 17722061

[b22] Nakano I. Joshi K. Visnyei K. Hu B. Watanabe M. Lam D. Wexler E. Saigusa K. Nakamura Y. Laks D. R. (2011). Siomycin A targets brain tumor stem cells partially through a MELK-mediated pathway. Neuro-oncol. 13, 622–634 10.1093/neuonc/nor023 21558073 PMC3107094

[b23] Nieuwkoop P. D. Faber J. (1994). Normal Table Of Xenopus Laevis (Daudin): A Systematical And Chronological Survey Of The Development From The Fertilized Egg Till The End Of Metamorphosis, 2nd edition New York, NY: Garland Publishing.

[b24] Paris J. Philippe M. (1990). Poly(A) metabolism and polysomal recruitment of maternal mRNAs during early Xenopus development. Dev. Biol. 140, 221–224 10.1016/0012-1606(90)90070-Y 2358121

[b25] Pickard M. R. Green A. R. Ellis I. O. Caldas C. Hedge V. L. M ourtada-Maarabouni M. Williams G. T. (2009). Dysregulated expression of Fau and MELK is associated with poor prognosis in breast cancer. Breast Cancer Res. 11, R60 10.1186/bcr2350 19671159 PMC2750122

[b26] Rappsilber J. Ishihama Y. Mann M. (2003). Stop and go extraction tips for matrix-assisted laser desorption/ionization, nanoelectrospray, and LC/MS sample pretreatment in proteomics. Anal. Chem. 75, 663–670 10.1021/ac026117i 12585499

[b27] Ryu B. Kim D. S. Deluca A. M. Alani R. M. (2007). Comprehensive expression profiling of tumor cell lines identifies molecular signatures of melanoma progression. PLoS ONE 2, e594 10.1371/journal.pone.0000594 17611626 PMC1895889

[b28] Saito R. Tabata Y. Muto A. Arai K. Watanabe S. (2005). Melk-like kinase plays a role in hematopoiesis in the zebra fish. Mol. Cell. Biol. 25, 6682–6693 10.1128/MCB.25.15.6682-6693.2005 16024803 PMC1190327

[b29] Schulze W. X. Mann M. (2004). A novel proteomic screen for peptide-protein interactions. J. Biol. Chem. 279, 10756–10764 10.1074/jbc.M309909200 14679214

[b30] Shevchenko A. Wilm M. Vorm O. Mann M. (1996). Mass spectrometric sequencing of proteins silver-stained polyacrylamide gels. Anal. Chem. 68, 850–858 10.1021/ac950914h 8779443

[b31] Swaminathan G. Cartwright C. A. (2012). Rack1 promotes epithelial cell-cell adhesion by regulating E-cadherin endocytosis. Oncogene 31, 376–389 10.1038/onc.2011.242 21685945

[b32] Tang V. W. (2006). Proteomic and bioinformatic analysis of epithelial tight junction reveals an unexpected cluster of synaptic molecules. Biol. Direct 1, 37 10.1186/1745-6150-1-37 17156438 PMC1712231

[b33] Tassan J. P. (2011). Cortical localization of maternal embryonic leucine zipper kinase (MELK) implicated in cytokinesis in early xenopus embryos. Commun. Integr. Biol. 4, 483–485 10.4161/cib.4.4.15669 21966578 PMC3181528

[b34] Tassan J. P. Le Goff X. (2004). An overview of the KIN1/PAR-1/MARK kinase family. Biol. Cell 96, 193–199 10.1016/j.biolcel.2003.10.009 15182702

[b35] Vulsteke V. Beullens M. Boudrez A. Keppens S. Van Eynde A. Rider M. H. Stalmans W. Bollen M. (2004). Inhibition of spliceosome assembly by the cell cycle-regulated protein kinase MELK and involvement of splicing factor NIPP1. J. Biol. Chem. 279, 8642–8647 10.1074/jbc.M311466200 14699119

[b36] Wehner P. Shnitsar I. Urlaub H. Borchers A. (2011). RACK1 is a novel interaction partner of PTK7 that is required for neural tube closure. Development 138, 1321–1327 10.1242/dev.056291 21350015

