# Supplementary material for: Cell-cycle dependent localization of MELK and its new partner RACK1 in epithelial versus mesenchyme-like cells in Xenopus embryo
Source: Biol Open. 2013 Aug 21;2(10):1037–48. doi: 10.1242/bio.20136080 (PMC3798187; doi:10.1242/bio.20136080)
Supplement: Supplementary Material [file supp_2_10_1037__index.html]

Cell-cycle dependent localization of MELK and its new partner RACK1 in epithelial versus mesenchyme-like cells in Xenopus embryo — Cell-cycle dependent localization of MELK and its new partner RACK1 in epithelial versus mesenchyme-like cells in Xenopus embryo — Supplementary Material 

# Cell-cycle dependent localization of MELK and its new partner RACK1 in epithelial *versus* mesenchyme-like cells in Xenopus embryo

## bio.20136080 Supplementary Material

**Files in this Data Supplement:**

- Supplementary Material - Isabelle Chartrain et al. doi: 10.1242/bio.20136080
