# Supplementary material for: Cell-cycle dependent localization of MELK and its new partner RACK1 in epithelial versus mesenchyme-like cells in Xenopus embryo
Source: Biol Open. 2013 Aug 21;2(10):1037–48. doi: 10.1242/bio.20136080 (PMC3798187; doi:10.1242/bio.20136080)
Supplement: Supplementary Material [file supp_bio.20136080_bio.20136080-s1.pdf]

Supplementary Material

Isabelle Chartrain et al. doi: 10.1242/bio.20136080

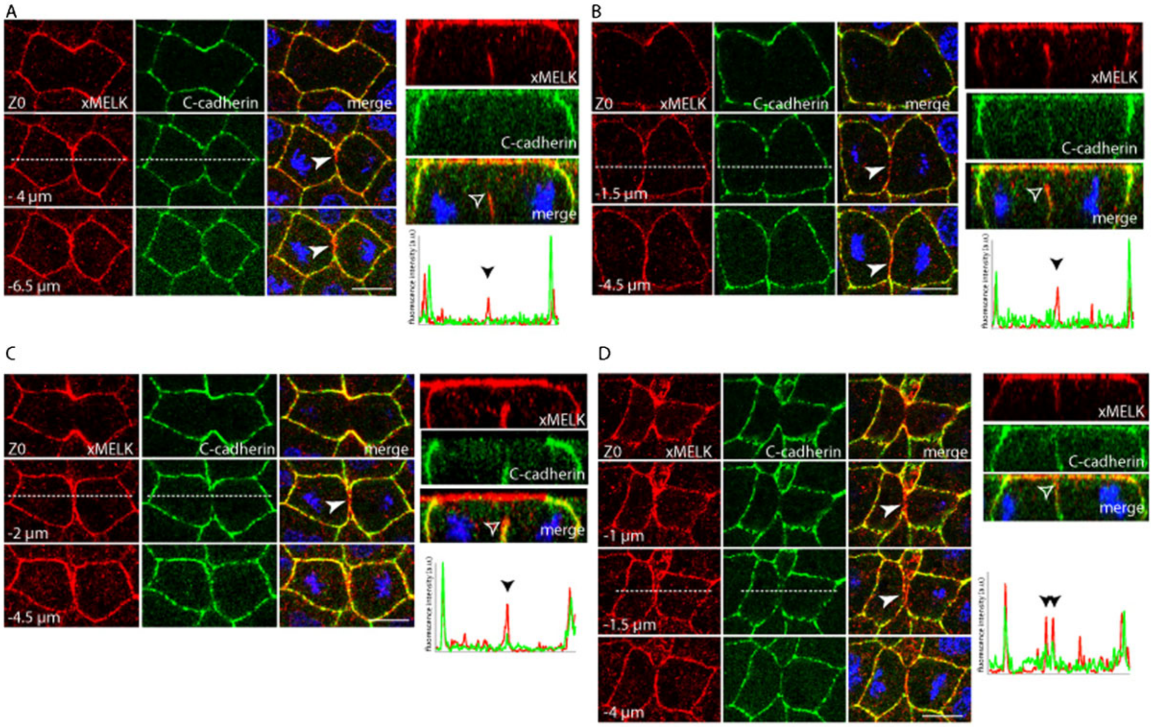

Fig. S1. Examples of representative indirect immunofluorescence images of gastrula epithelial cells showing that xMELK but not C-cadherin localizes at the cytokinetic furrowing tip. Indirect immunofluorescence with anti-xMELK (red) and anti-C-cadherin (green) antibodies was performed on fixed Xenopus embryos at gastrula stage as in Fig. 2A. DNA was stained with TO-PRO-3 (blue). Images were merged to visualize co-localization of xMELK with C-cadherin (merge). Single confocal planes are shown. Arrowheads point to xMELK accumulated at cytokinetic furrow tip. Empty arrowheads on orthogonal projection point on the cytokinetic tip where xMELK is present without C-cadherin. Coloured curves show the fluorescence intensities for xMELK (red) and C-cadherin (green) along the dashed lines drawn on confocal images. Black arrowheads indicate the position of the cytokinetic furrows. Note that in panel D a gap is observed between the two daughter cells. Scale bars: 10 µm.

Biology Open

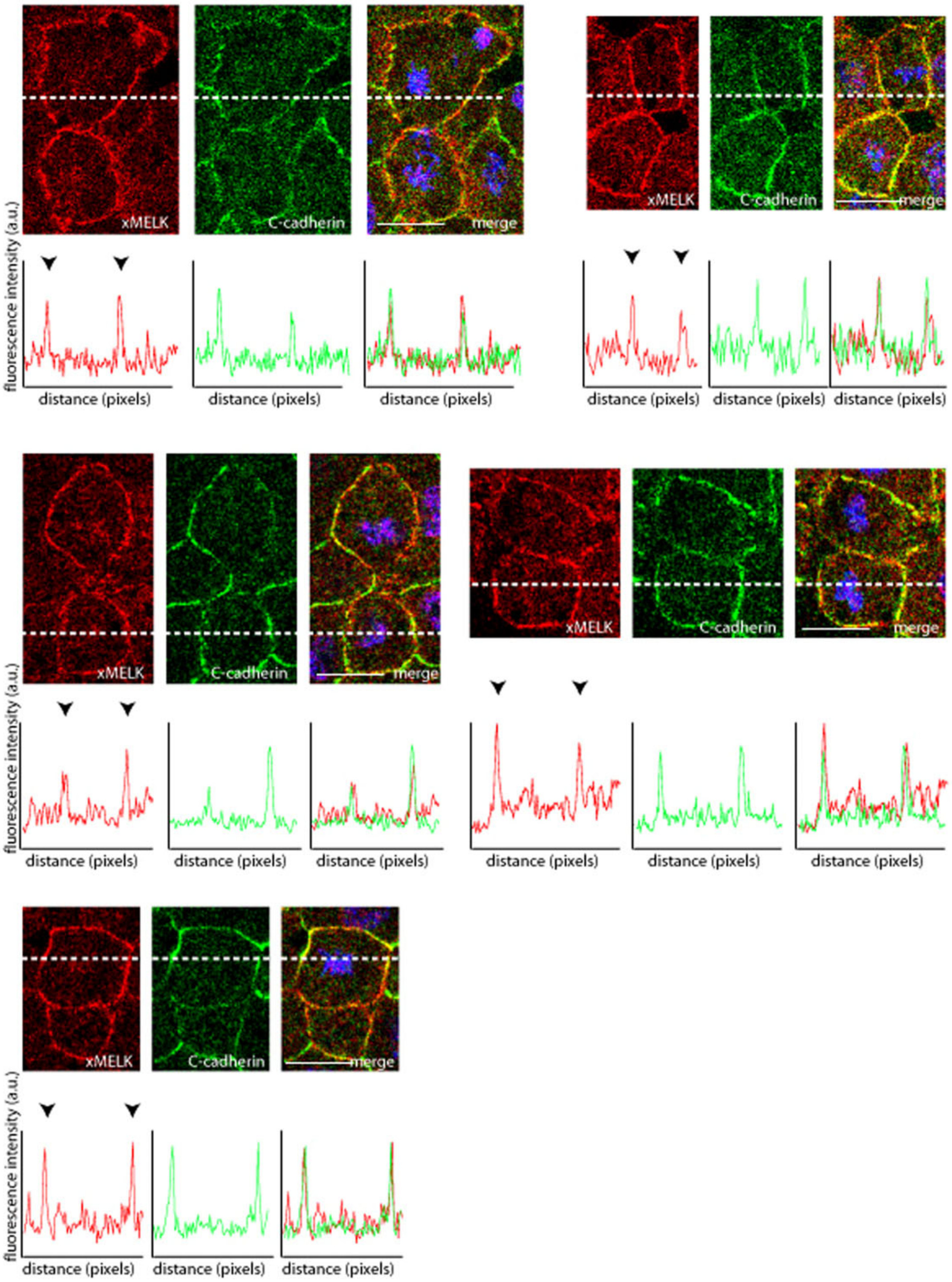

Fig. S2. Examples of representative indirect immunofluorescence images of gastrula mesenchymal-like cells showing that xMELK accumulates at the periphery of dividing cells. Indirect immunofluorescence with anti-xMELK (red) and anti-C-cadherin (green) antibodies was performed on fixed Xenopus embryos at gastrula stage as in Fig. 2B. DNA was stained with TO-PRO-3 (blue). Images were merged to visualize co-localization of xMELK with C-cadherin (merge). Asterisks indicate dividing cells. A single confocal plane is shown. Coloured curves show the fluorescence intensities for xMELK (red) and C-cadherin (green) along the dashed lines drawn on confocal images. Arrowheads point to xMELK accumulated at the cell periphery in dividing cells. Scale bars: 10 µm.

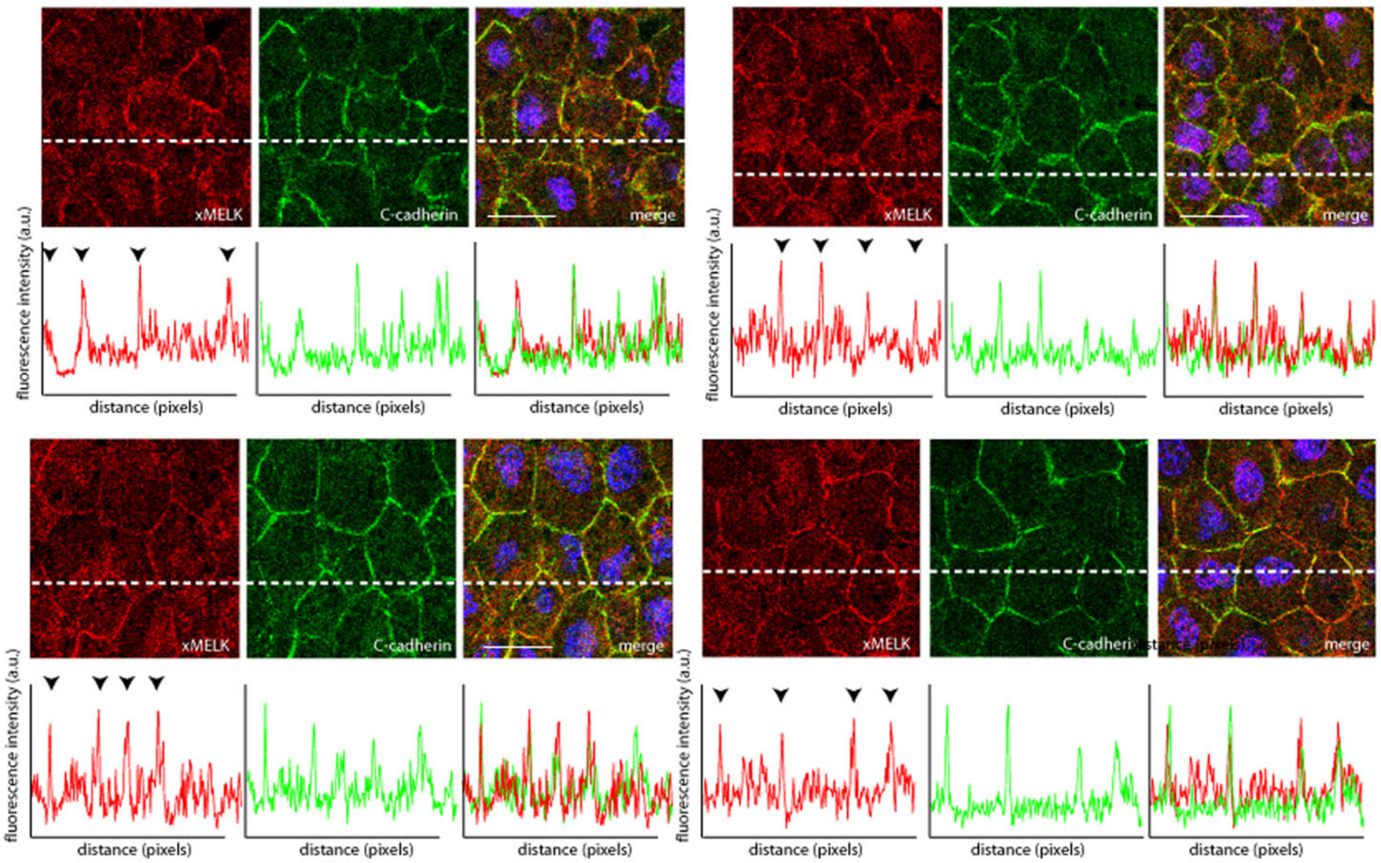

Fig. S3. Examples of representative indirect immunofluorescence images of gastrula mesenchymal-like cells showing that xMELK accumulates at the periphery of interphase cells. Indirect immunofluorescence with anti-xMELK (red) and anti-C-cadherin (green) antibodies was performed on fixed Xenopus embryos at gastrula stage as in Fig. 2B. DNA was stained with TO-PRO-3 (blue). Images were merged to visualize co-localization of xMELK with C-cadherin (merge). Asterisks indicate dividing cells. A single confocal plane is shown. Coloured curves show the fluorescence intensities for xMELK (red) and C-cadherin (green) along the dashed lines drawn on confocal images. Arrowheads point to xMELK accumulated at the cell periphery in interphase cells. Scale bars: 10 µm.

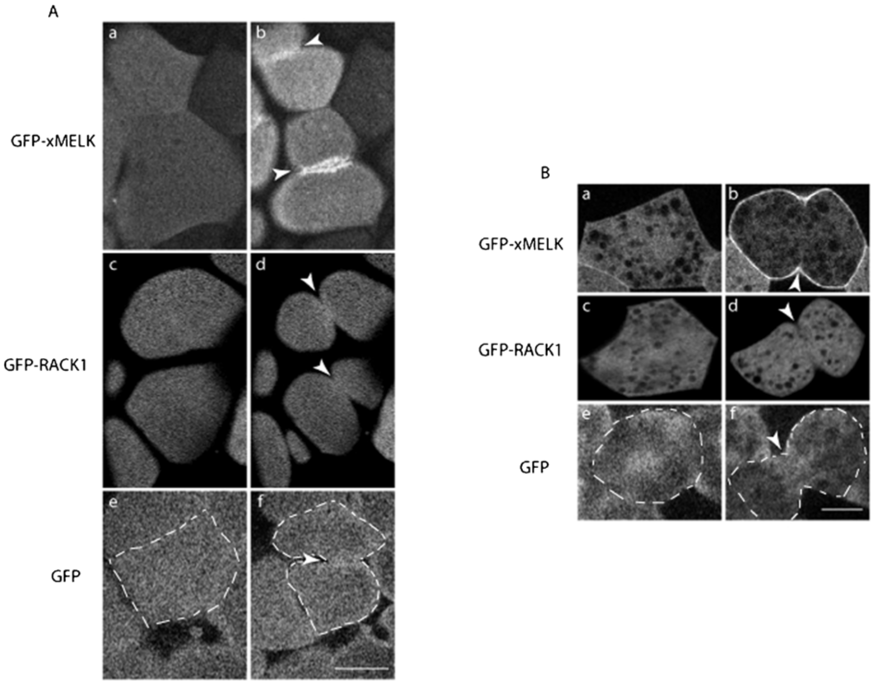

Fig. S4. Contrary to GFP-xMELK KR, GFP-RACK1 does not accumulate at the cytokinetic furrow and the cell cortex during cytokinesis in blastula and gastrula embryos. Localization of GFP-xMELK KR (an inactive xMELK mutant unable to induce cytokinesis defects) (Le Page et al., 2011), GFP-RACK1 and GFP alone in blastula (A) and gastrula (B) embryos. A single confocal plane of cells before (Aa,c,e and Ba,c,e) and during cytokinesis (Ab,d,f and Bb,d,f) is shown. GFP-xMELK KR accumulates at the division furrow and the cell cortex in cytokinetic cells of blastula (Ab) and at the cell cortex in gastrula cells (Bb). In contrast, in both blastula and gastrula embryos, GFP-RACK1 (Ad and Bd, respectively) and GFP alone (Af and Bf, respectively) do not show this localization. Arrowheads point to the cytokinetic furrows. Dashed lines indicate dividing cells. Scale bars: 100 µm (A), 10 µm (B).

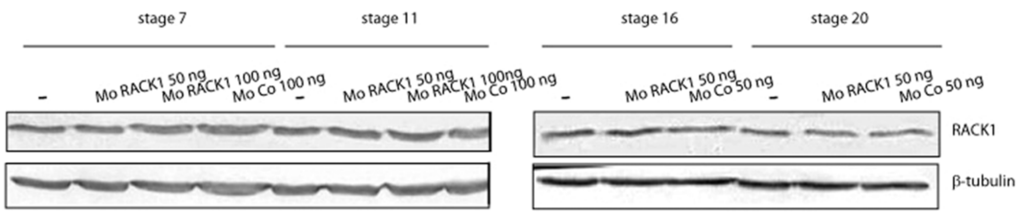

Fig. S5. The endogenous maternal pool of RACK1 protein was not affected by Morpholinos in the early embryo. Embryos at the two-cell stage were either not injected (–) or injected in the two blastomeres with anti-RACK1 Morpholinos (Mo RACK1: CCCGAAGTGTCATTTGCTCAGTCAT, the total amount injected was 50 ng and 100 ng) or with the control Morpholino (MoCo from Gene Tools, the total amount injected was 50 ng and 100 ng, respectively). The embryos were harvested at stage 7, 11, 16 and 20 (Nieuwkoop and Faber, 1994). Total proteins were extracted and analyzed by Western blotting with anti-RACK1 antibody and anti-beta-tubulin used as a loading control.

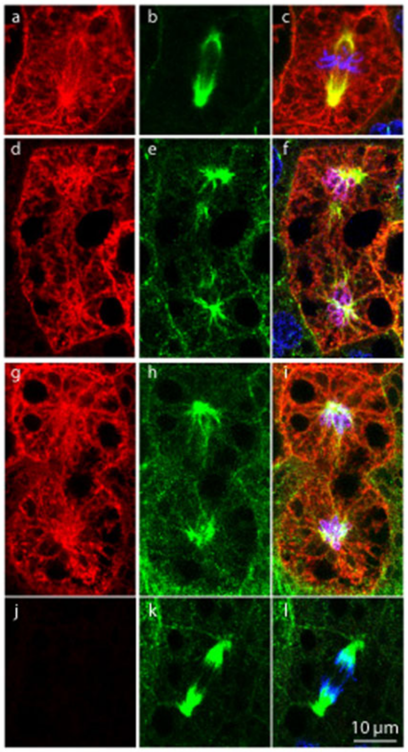

Fig. S6. FLAG-RACK WD5–7 localizes on microtubules in gastrula embryos. Gastrula embryos expressing FLAG-RACK1 WD5–7 (a–i) were fixed and processed for indirect immunofluorescence as in Fig. 8A. The FLAG-RACK1 WD5–7 protein was detected with a rabbit polyclonal anti-FLAG (Sigma, 1:200, a,d,g) and tubulin with a mouse monoclonal anti-tubulin (TUB 2.1, Sigma, 1:200, b,e,h) antibody. Images were merged to visualize co-localization of FLAG-RACK1 WD5–7 with microtubules (c,f,i). DNA is in blue. Uninjected embryos used as negative controls (j,k,l) were processed as FLAG-RACK1 WD5–7 expressing embryos. Scale bar: 10 µm.

Biology Open
